# Enhancing the Minimum Awareness Failure Distance in V2X Communications: A Deep Reinforcement Learning Approach

**DOI:** 10.3390/s24186086

**Published:** 2024-09-20

**Authors:** Anthony Kyung Guzmán Leguel, Hoa-Hung Nguyen, David Gómez Gutiérrez, Jinwoo Yoo, Han-You Jeong

**Affiliations:** 1 Department of Electrical Engineering, Pusan National University, Busan 46241, Republic of Korea; kyung.guzman@pusan.ac.kr (A.K.G.L.); nguyenhoahungit@gmail.com (H.-H.N.); 2 Intelligent Systems Research Lab, Intel Labs, Intel Tecnología de México, Jalisco 45017, Mexico; david.gomez.g@ieee.org; 3 Unidad Académica Zapopan, Instituto Tecnológico José Mario Molina Pasquel y Henríquez, Tecnológico Nacional de México, Jalisco 45019, Mexico; 4 Department of Automobile and IT Convergence, Kookmin University, Seoul 02707, Republic of Korea; jwyoo@kookmin.ac.kr; 5 Robotic Institute of Non-Destructive In-Line Inspection (RiNDi), Pusan National University, Busan 46241, Republic of Korea

**Keywords:** vehicle-to-everything (V2X) communications, beaconing, deep reinforcement learning, vehicle awareness, minimum awareness failure distance

## Abstract

Vehicle-to-everything (V2X) communication is pivotal in enhancing cooperative awareness in vehicular networks. Typically, awareness is viewed as a vehicle’s ability to perceive and share real-time kinematic information. We present a novel definition of awareness in V2X communications, conceptualizing it as a multi-faceted concept involving vehicle detection, tracking, and maintaining their safety distances. To enhance this awareness, we propose a deep reinforcement learning framework for the joint control of beacon rate and transmit power (DRL-JCBRTP). Our DRL−JCBRTP framework integrates LSTM-based actor networks and MLP-based critic networks within the Soft Actor-Critic (SAC) algorithm to effectively learn optimal policies. Leveraging local state information, the DRL-JCBRTP scheme uses an innovative reward function to increase the minimum awareness failure distance. Our SLMLab-Gym-VEINS simulations show that the DRL-JCBRTP scheme outperforms existing beaconing schemes in minimizing awareness failure probability and maximizing awareness distance, ultimately improving driving safety.

## 1. Introduction

The advent of Cooperative Intelligent Transportation Systems and connected vehicles has revolutionized transportation by facilitating seamless vehicle-to-everything (V2X) communications between vehicles and various road infrastructure components. This cooperative framework significantly enhances road safety and plays a pivotal role in improving traffic flow and overall transportation efficiency [1,2,3,4,5].

**Figure 1:**
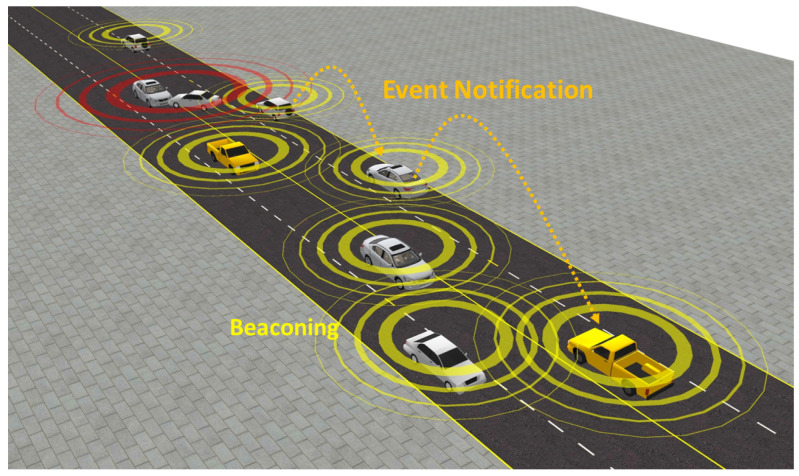
Example of beaconing and event notification.

Information sharing in V2X safety applications is primarily categorized into beaconing and environment notification, as shown in Figure 1. Beaconing, executed via the Cooperative Awareness Messages [3] and the Basic Safety Messages [4], enables vehicles to continuously share critical kinematic information, such as position, velocity, acceleration, and heading. On the other hand, environment notifications, relaying the Distributed Environment Notification Messages [5], are designed to inform upstream vehicles about impending hazards or critical events within a specified region of interest, thereby providing timely warnings that enable drivers to take appropriate actions to avoid accidents and enhance situational awareness. Our research focuses on cooperative awareness via beaconing, emphasizing its critical importance in preventing vehicle-to-vehicle (V2V) collisions through the accurate and immediate sharing of information among neighboring vehicles.

Despite the extensive research on the beaconing problem over the past two decades, there is still no consensus on the precise definition of awareness in V2X communications [6,7,8,9,10,11,12,13,14,15,16,17,18,19,20]. Typically, awareness is viewed monolithically as a vehicle’s ability to perceive and share real-time kinematic information about itself and neighboring vehicles to enhance safety and efficiency [1,2]. Based on this view, the initial approaches either control transmit power (TP) [6] or beacon rate (BR) in [7,8,9,10,20] to maintain an appropriate beacon load in dynamic vehicular environments. Later, these approaches are extended to a joint approach with BR and TP in [11,12,13,14]. However, all these approaches focus on access-layer performance neglecting the key requirements of vehicle awareness. Some approaches have used direct [17] or indirect [15,16] tracking metrics to measure discrepancies between the actual and estimated kinematics of vehicles. While these schemes help ensure that surrounding vehicles are adequately tracked, they fail to consider neighbor vehicles that do not receive any beacon messages, which poses a significant threat to driving safety by potentially leaving undetected vehicles unaccounted for in the V2V collision avoidance process.

To address these limitations, we argueHowever, we propose that awareness should be understood as a multi-faceted concept involving vehicle detection, vehicle tracking, and the minimum awareness failure distance (mAFD), defined as the shortest distance within which all neighbor vehicles are detected and successfully tracked. The mAFD is crucial as it represents the closestThe mAFD represents the shortest distance to any undetected or untracked vehicles that pose a high risk of V2V collisions. By this definition, increasing the mAFD is essential for enhancing driving safety.

To achieve this, we propose a novel deep reinforcement learning (DRL) framework for the joint control of beacon rate and transmit power (DRL-JCBRTP) that models beaconing as a Markov decision process (MDP). Based on locally observable state information, a vehicle (agent) determines its BR and TPbeacon rate and transmit power (action) to optimize our intricately crafted reward functions (return), considering factors such as awareness, beacon aging, and channel congestion. This DRL framework is applicable to any model-free DRL scheme, making it a versatile tool for enhancing beaconing performance. We employ the Soft Actor-Critic (SAC) [21] algorithm due to its sample efficient learning and stability. Furthermore, we present and analyze different neural network (NN) architectures within the SAC framework to evaluate their impact on the performance of the DRL-JCBRTP scheme. The contributions of this paper are summarized as follows:

- We present a new definition of awareness in V2X communications, framing it as a multi-faceted concept involving vehicle detection, tracking, and safe distances.
- We propose the DRL−JCBRTP framework, which integrates LSTM-based actor networks and MLP-based critic networks within the SAC framework, enabling the effective learning of optimal policies.a model-free DRL approach to improve cooperative vehicle awareness in V2X communications.
- We design an innovative reward function that leverages local state information to meticulously address multiple aspects of vehicle awareness.
- Based onWe develop our SLMLab-Gym-VEINS simulation framework, we to demonstrate the superior performance of the DRL−JCBRTP scheme in terms of the probability and distance of awareness failure, which ultimately improves driving safety.

The remainder of the paper is organized as follows. Section 2 outlines the related works on beaconing. Our definition of awareness in V2X communications is presented in Section 3. Then, Section 4 presents our DRL−JCBRTP framework in detail. The numerical results of the DRL−JCBRTP scheme are compared with the existing beacon schemes in Section 5. Finally, we conclude this paper in Section 6.

## 2. Related Works

Beaconing is the process of regularly broadcasting the kinematics of sending a vehicle to prevent V2V collisions. Two key parameters of beaconing are the BR and TPbeacon rate (BR) and transmit power (TP). The BR refers to the frequency at which these broadcast messages are sent, typically measured in Hertz (Hz), while TP determines the range over which the beacon messages can be effectively received. Beaconing faces two major challenges: updating vehicle kinematics promptly for better awareness under unreliable wireless channels, and maintaining an appropriate channel busy ratio (CBR) to avoid both channel underutilization and excessive message collisions.

**Figure 2:**
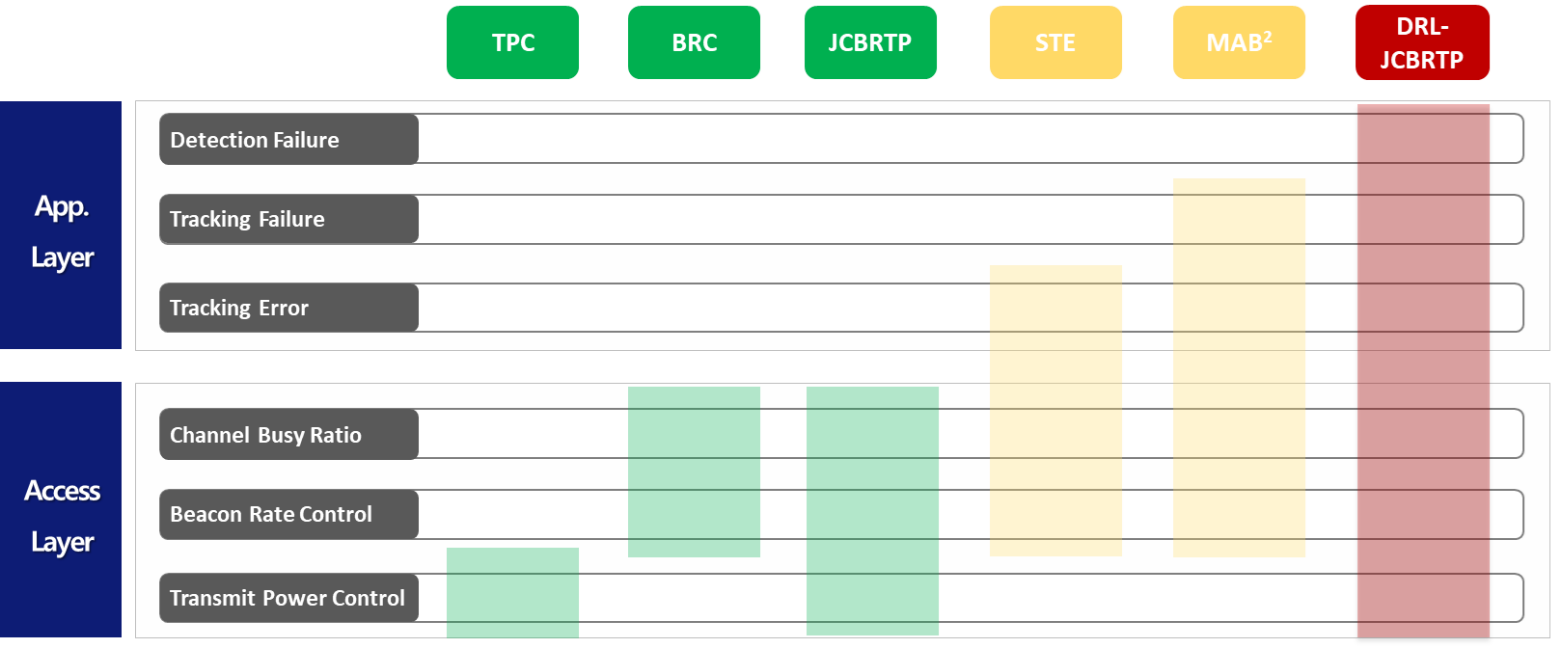
Comparison of beaconing approaches: TPC [6], BRC [7,8,9,10], JCBRTP [11,12,13,14], STE [15,16], and DRL-JCBRTP. (Green: Access-layer approaches, Yellow: Partial cross-layer approaches, and Red: Full cross-layer approaches).

Although the beaconing problem has been extensively studied in the last two decades [6,7,8,9,10,11,12,13,14,15,16,17,18,19,20], these studies do not fulfill the safety requirements of safety applications, as shown in Figure 2. The seminal work in [6] presents a fair TPtransmit power control algorithm, ensuring thatto fairly control the beacon TP so that the beacon load within a two-hop distance is limited to a given threshold. However, we believe that providing equal channel access opportunities without considering the varyingvehicle kinematics of vehicles may not achieve true fairness atin the application layer.

To address the challenge of channel congestion through BR control, a few BRbeacon rate control (BRC) schemes have been proposed in the literature [7,8,9,10]. A new adaptive BRC algorithm is proposed to ensure fair and efficient channel utilization in [7]. The authors in [8] present a simple algorithm to determine the beacon interval for stable CBR against dynamic beacon loads. A priority-based scheme increases the BR for vehicles with increased collision risks in terms of time-to-collision [9]. A game theoretic approach in [10] modifies the BR using risk factors, aiming to improve adaptability and safety. An reinforcement learning (RL) approach redefines BRC as an optimal decision-making problem, leveraging a Q-table and function approximation to make decisions based on the current BR and vehicle density [20]. A common limitation of BRC approaches is their absence of TP control, which can hinder their ability to effectively maintain the CBR within an optimal range.

A JCBRTP approach is proposed to efficiently control the channel load by jointly managing the BR and TPbeacon rate and power [11,12,13,14]. The ETSI decentralized congestion control scheme in [11] jointly controls the BR, TP, data rate, and receiver sensitivity to keep the CBR in a desirable range. A non-cooperative game-theoretic approach, called the beacon frequency and power control (BFPC) scheme, is proposed in [12] to find the Nash equilibrium of BR and TP in a distributed manner. In addition, the Markov decision process with a rate and power (MDPRP) framework employs Q-learning to derive the optimal policy for BR and TP that keeps the CBR in a desirable range [13]. Furthermore, the paper in [14] addresses the joint power-rate optimization problem for the Rayleigh fading channel by solving its Lagrangian dual problem. Although improving the access-layer performance indirectly enhances awareness, a key limitation of the BRC approach is its weak connection to application-layer requirements, such as the detection and tracking of neighbor vehicles.

Assuming that each vehicle has a remote kinematics estimator to estimate the kinematics of each sending vehicle based on its most recently received beacon information, the beaconing schemes in [15,16]suspected tracking error (STE) approaches address the above limitations by devising an indirect trackingaverage metric, called the suspected tracking error (STE)STE, to assess the average tracking error between the real and estimated kinematics of the sending vehicle. These schemesThe beaconing schemes in [15,16] control the BR to reduce the STE metric while maintaining the CBR within a desirable range. However, theythe STE approaches do not account for the variation in tracking errors among neighbor vehicles, which often fails to limit their tracking errors to a tracking threshold $E_{T}$.

On the other hand, the mobility-adaptive beacon broadcast (MAB^2^) scheme guarantees a successful tracking of the sending vehicle to a subset of its neighbor vehicles, which successfully receive at least one beacon out of the most recent *K* beacons, which is called the guaranteed tracking of degree *K* [17]. Despite its theoretically proven optimality in channel bandwidth usage, timely dissemination of beacons, and low tracking failure probability, the MAB^2^ scheme encounters challenges in refreshing kinematic information and managing TP, indicating a potential area for further improvement.

To the best of our knowledge, this work is the first research to comprehensively address all of the awareness requirements of the beaconing problem in V2X communications, including vehicle detection, tracking, and the mAFD. To address this problem, we adopt the DRL approach because it allows for real-time learning and optimization of policies in complex, dynamic environments, crucial for ensuring the safety and efficiency of V2X communications. Although there are a few DRL approaches in [13,18,19,20], they typically focus on the control of channel congestion by controlling data rate [18,19,20], BR [13], or TP [13,18]. In contrast, our DRL−JCBRTP simultaneously addresses vehicle detection, tracking, and maintaining the mAFD, providing a more holistic solution to the beaconing problem in V2X communications.

## 3. Our Definition of Awareness in V2X Communications

Although a plethora of beaconing approaches in [6,7,8,9,10,11,12,13,14,15,16,17,18,19,20] have been presented in the last two decades, it is surprising that there is almost no consensus in both academia and industry on the precise definition of awareness in V2X communications. Instead, it is conventionally understood as the ability of a vehicle to continuously perceive and share real-time information about its own kinematic state and those of its neighbor vehicles to enhance driving safety and traffic efficiency [1,2]. However, we argue that awareness cannot be explained by such a monolithic view; it must be understood as a multi-faceted concept that comprehensively considers vehicle detection, tracking, and maintaining a safe distance, all of which are crucial for ensuring driving safety and traffic efficiency.

Given neighbor vehicles in the transmission range $D_{T}$, the goal of this section is to present our definition of awareness in terms of three key components:

- **Vehicle detection** refers to the ability to recognize the presence of a neighbor vehicle. If the ego vehicle receives at least one beacon from a neighbor vehicle in $D_{T}$, the neighbor vehicle is considered successfully detected; otherwise, its detection is deemed to have failed.
- **Vehicle tracking** involves continuously monitoring and updating the kinematic states of a neighbor vehicle. If the tracking error is within threshold $E_{T}$, the neighbor vehicle is considered successfully tracked; otherwise, its tracking is considered to have failed.
- **Minimum awareness failure distance (mAFD)** is the distance between the ego vehicle and the nearest neighbor vehicle that has failed to be detected or tracked. Increasing the mAFD is crucial for maintaining road safety and preventing accidents.

Notice that each neighbor vehicle can be in one of the following three states: *undetected* (vehicle detection failure), *untracked* (successful vehicle detection but tracking failure), and *tracked* (successful vehicle tracking).

**Figure 3:**
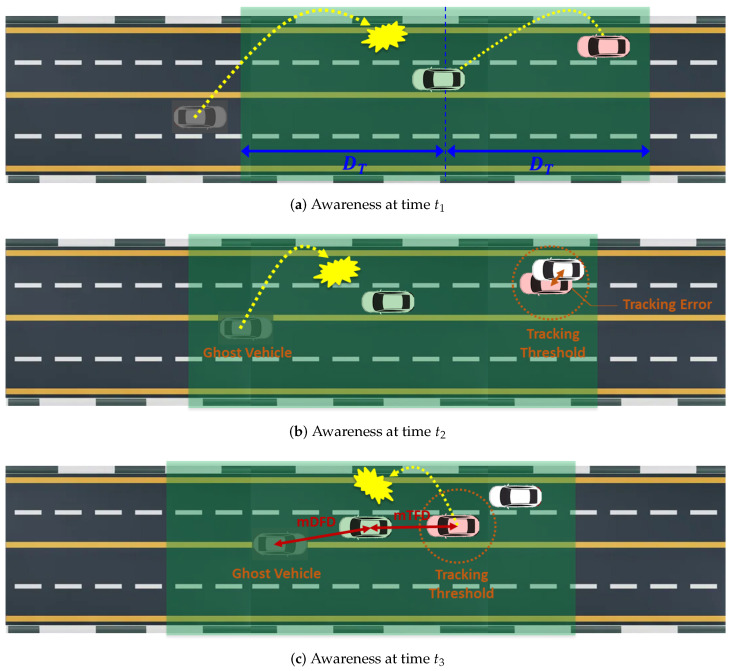
Awareness of ego vehicle (green) at three timepoints ($t_{1}<t_{2}<t_{3}$).

To further clarify the definition of mAFD, we present an example of the awareness of the green-colored ego vehicle at times $t_{1}$, $t_{2}$, and $t_{3}$ ($t_{1}<t_{2}<t_{3}$), as illustrated in Figure 3. The transmission range $D_{T}$ is represented by a green transparent area. At time $t_{1}$, the ego vehicle successfully receives the beacon from pink-colored neighbor vehicle 1 (NV1), while it fails to receive the beacon from gray-colored neighbor vehicle 2 (NV2), as shown in Figure 3a. Then, the initial states of NV1 and NV2 at time $t_{1}$ are tracked and undetected, respectively. Figure 3b shows that the states of NV1 and NV2 at time $t_{2}$ remain unchanged from time $t_{1}$. This is due to the ego vehicle’s failure to receive a new beacon from NV2 and because the tracking error between NV1 and estimated NV1 (white vehicle) remains within the tracking threshold $E_{T}$. At time $t_{3}$, the state of NV1 changes to untracked because a new beacon from NV1, containing the lane-changing behavior, is not successfully received at the ego vehicle. Here, we devise three awareness performance measures, as follows:

- **Minimum detection failure distance (mDFD)** is the distance to the nearest undetected vehicle. This measure helps assess the critical proximity at which the ego vehicle is unaware of nearby vehicles, posing the most significant safety risk.
- **Minimum tracking failure distance (mTFD)** is the minimum distance to the nearest untracked vehicle. This measure evaluates the risk associated with vehicles that, although detected, have their kinematic information inaccurately monitored, potentially leading to unsafe driving maneuvers.
- **Maximum tracking error (MTE)** is the largest tracking error over all tracked neighbor vehicles. While it does not pose a risk of accidents, this measure indicates the upper bound of the tracking inaccuracy of tracked neighbor vehicles.

Finally, the mAFD is given by the shorter distance between the mDFD and the mTFD. This measure is crucial because it represents the closest distance to which the ego vehicle might fail to be aware of a neighbor vehicle due to failure in detection or inaccurate tracking. As such, increasing the mAFD is vital for ensuring that the ego vehicle maintains a safe buffer from undetected or poorly tracked vehicles, thereby significantly reducing the risk of collisions and enhancing overall driving safety.

Based on our novel awareness measures, the following section introduces the DRL-JCBRTP framework that models the beaconing as an optimal decision-making problem.

## 4. Our DRL−JCBRTP Framework

This section briefly describes our system model, followed by a general overview of the DRL framework and the detailed design principles of our DRL-JCBRTP scheme.

### 4.1. System Model

In this section, we evolve the system model of the MAB^2^ scheme in [17] for integration with our DRL framework. Basically, our system model can interact with multiple access-layer technologies, for example, the DSRC/WAVE protocol suite [22,23,24] and/or cellular V2X technologies [25,26,27]. This is because the beaconing agent determines the high-level parameters of the access layer, such as the rate of the beacon broadcast and the TP to cover a given transmission range $D_{T}$, that can be adapted to the underlying access technologies. In our model, time is segmented into a series of frames {n} with duration *T*. It is assumed that vehicle kinematics, including position and speed at frame *n*, are sampled at the beginning of each frame, marked as t=nT. This beacon information is subsequently broadcast to neighbor vehicles during the frame period, t∈[nT,(n+1)T).

**Figure 4:**
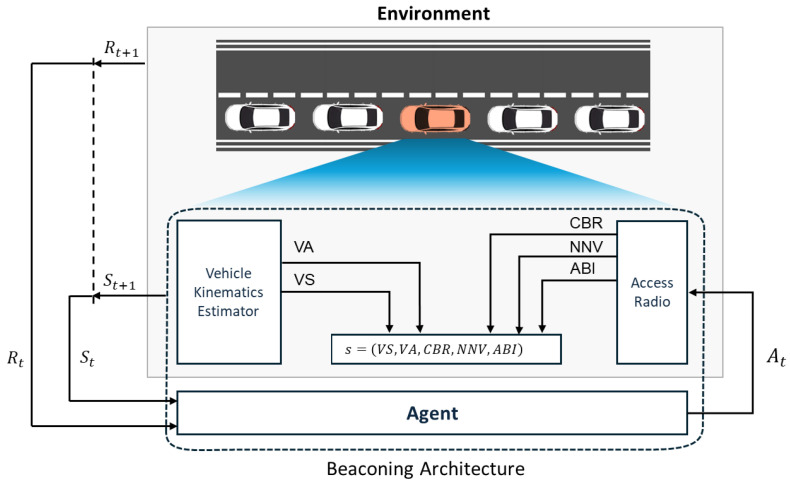
Architecture of our beaconing agent in an MDP framework.

Figure 4 shows the architecture of a beaconing agent in an MDP framework. Due to a time-varying and lossy wireless channel, a neighbor vehicle may not receive the latest beacons of the sending vehicle. If none of the past beacons from the sending vehicle is successfully received by a neighbor vehicle within $D_{T}$, the sending vehicle becomes a ghost vehicle (detection failure) in the V2X communication framework, as shown in Figure 3b,c. On the other hand, if at least one beacon is successfully received by a neighbor vehicle, it belongs to one of the (K+1) vehicle groups $V=\{V_{1},\;V_{2},\;\cdots ,\;V_{K},\;V_{K+}\}$. Vehicle group $V_{k}$ represents a subset of neighbor vehicles whose latest beacon is the *k*-th previous beacon of the sending vehicle. In our DRL-JCBRTP framework, we consider the awareness of a neighbor vehicle in the first *K* vehicle groups $V_{G}=V-V_{K+}$ only, excluding vehicle group $V_{K+}$ due to its severe wireless channel conditions. *K* constant-speed position estimators are assumed to be used as the remote kinematics estimators that predict the current position of each sending vehicle in $V_{G}$. Then, the *tracking error (TE)* of a neighbor vehicle in $V_{k}$ is formally defined as the Euclidean distance from the actual position of the sending vehicle to the position of estimator *k*. If the TE of a neighbor vehicle exceeds a tracking threshold $E_{T}$, then its kinematics estimation is considered a *tracking failure* (See Figure 3c).

### 4.2. DRL−JCBRTP NN Architecture Design Based on SAC Algorithm

RL involves optimizing actions for an agent to maximize cumulative discounted rewards, typically modeled by MDPs [28]. In this context, the policy π(a|s), value function $V_{\pi }\left(s\right)$, and action-value function $Q_{\pi }\left(s,a\right)$ are central concepts, with the goal being to learn an optimal policy $\pi^{*}$ that maximizes expected return.

RL categorizes control strategies into model-based and model-free mechanisms [28]. Model-based control requires agents to learn a model of the environment’s dynamics and rewards for action planning, offering efficiency in terms of samples but depending on the model’s accuracy. Conversely, model-free control bypasses the need to model environmental dynamics by learning directly from experience. This framework is further divided into on-policy and off-policy methods based on their policy update and evaluation mechanisms [28]: on-policy methods adjust policies during their execution, while off-policy methods aim to identify an optimal policy through exploration with a distinct policy.

DRL extends RL using deep learning techniques, which are particularly useful in high-dimensional environments [29], and leverages deep NNs (DNNs) for function approximation, enabling direct learning from raw sensory data and the ability to handle extensive state spaces. Considering the environment model is not sufficiently defined, our DRL−JCBRTP framework adopts a model-free, off-policy SAC algorithm that balances exploration and exploitation via entropy maximization [21]. SAC uses actor and critic NNs for policy and value function approximation, respectively.

**Actor NN**: The policy objective in the reparameterized form within the actor NN is

(1) $$J_{\pi }\left(\phi \right)=E_{s_{t}∼D,\epsilon_{t}∼N}\left[\alpha \log\pi_{\phi }\left(f_{\phi }\left(\epsilon_{t};s_{t}\right)|s_{t}\right)-Q_{\theta }\left(s_{t},f_{\phi }\left(\epsilon_{t};s_{t}\right)\right)\right],$$

where the expectation of state $s_{t}$ is sampled from the replay buffer D, α is the temperature parameter to balance between exploration and exploitation, and $f_{\phi }\left(\epsilon_{t};s_{t}\right)$ is the action from NN given sampled noise $\epsilon_{t}$ from a normal distribution N and state $s_{t}$. The first term in the expectation represents the entropy of the action under the current policy $\pi_{\phi }$, and the second term is the expected return associated with the chosen state-action pair.

In the existing SAC algorithms, the actor NN typically uses a multi-layer perceptron (MLP) to generate actions at each time step, selecting the optimal action within the given action space [21,29]. This approach is effective in environments where actions are determined based on immediate observations. However, in our beaconing problems, where the goal is to control the BR, the challenge extends to predicting and adjusting beacon intervals over time, requiring the modeling of temporal dependencies. Since BRC is fundamentally a time-series prediction problem, this paper deviates from the traditional use of MLPs and employs recurrent NNs (RNNs) to generate actions. Specifically, Long Short-Term Memory (LSTM) networks are introduced within the actor NN to capture and leverage sequential patterns in beaconing data. The RNN-based actor NN is designed to predict future beacon intervals by considering the temporal context provided by previous actions and states, enabling more accurate and adaptive control in dynamic environments. By integrating RNNs into the SAC framework, the proposed method directly addresses the time-dependent nature of the beaconing task, significantly enhancing the actor NN’s ability to manage sequential data and ultimately improving the efficiency and safety of V2X communications.

**Critic NN**: The critic network is updated by minimizing the mean square error between the expected target Q value $r\left(s_{t},a_{t}\right)+\gamma {\mathrm{error}}_{\left(s_{t+1}\right)∼p}\left[V_{\bar{\theta }}\left(s_{t+1}\right)\right]$ and the current Q-value $Q_{\theta }\left(s_{t},a_{t}\right)$ predicted by the critic network, i.e.,

(2) $$J_{Q}\left(\theta \right)=E_{\left(s_{t},a_{t}\right)∼D}\left[\frac{1}{2}{\left(Q_{\theta }\left(s_{t},a_{t}\right)-\left(r\left(s_{t},a_{t}\right)+\gamma E_{\left(s_{t+1}\right)∼p}\left[V_{\bar{\theta }}\left(s_{t+1}\right)\right]\right)\right)}^{2}\right],$$

where the expectation is taken over a minibatch of state-action pairs ($s_{t},a_{t}$) sampled from the replay buffer D, $r(s_{t},a_{t})$ is the immediate reward received after taking action $a_{t}$ in state $s_{t}$, γ is the discount factor, and the nested expectation is on the next states $s_{t+1}$ sampled from the environment’s dynamics *p*.

The critic NN in our DRL-JCBRTP employs the MLP to approximate the value function due to its capability to model complex, non-linear relationships between states and actions. The MLP enables the critic NN to accurately approximate the action-value function $Q_{\theta }\left(s_{t},a_{t}\right)$, which is essential for evaluating the expected return of different state-action pairs. By leveraging the MLP in the critic NN, the SAC algorithm can accurately assess the value of actions proposed by the actor NN, thereby facilitating the learning of robust policies that optimize long-term rewards in V2X communication scenarios.

### 4.3. MDP Specifications of Our DRL-JCBRTP

MDPs provide a mathematical framework for sequential decision-making, where outcomes are influenced by the decision-maker’s policy, making them highly suitable for the intricate beaconing dynamics. Utilizing this framework, an RL agent can effectively adjust the BR and TP to improve awareness and to maintain an appropriate channel load. The Markov property simplifies the decision-making process by associating future outcomes directly with the current state, eliminating reliance on past decisions. This framework is essential for developing strategies that adapt to present conditions, providing a cohesive approach to the challenges of beaconing. Our MDP formulation embodies this principle, emphasizing policy-driven decisions for the immediate scenario, with the specifics outlined as follows:

1. *Agent*, tasked with learning the optimal policy and executing actions, is represented by individual vehicles in the road environment. These vehicles observe their local state information to adjust their BR and TP accordingly, aiming to fulfill diverse objectives. This setup fosters a decentralized framework, where every vehicle independently makes a decision informed by its specific state information. Despite the decentralized nature of decision-making, we assume that the same underlying policy is used across all vehicles, ensuring a coherent and unified strategy for managing BR and TP on the road.
2. *Environment* serves as the complex and dynamic setting in which the agent operates, providing critical feedback on the agent’s actions and influencing its subsequent state. In addition, it plays a pivotal role in delivering essential state information and issuing rewards or penalties based on the agent’s decisions. This feedback loop is essential for steering the agent toward optimal behaviors, enabling it to adeptly adjust to and manage the continuously changing road and traffic conditions.
3. *Actions*, allowing the agent to react to the environment, are designated as $A_{t}=a\in A$, where A is the set of possible actions. At each frame, the agent evaluates its current state to decide on a beacon broadcast, thus affecting the BR and TP. These actions are structured as a pair a=(b,p) consisting of the decision to transmit a beacon (*b*) and the TP (*p*), where b={0,1} indicates whether to broadcast a beacon frame (1) or not (0), and if b=1, an appropriate TP *p* dBm is chosen to mitigate channel congestion. Notably, all these actions conform to access-layer standards, ensuring regulatory compliance [22,24,26,27].
4. The *state* is a vector s∈S consisting of the internal parameters of the agent and its observation of the environment, where *S* represents the complete set of potential states. This state, forming the critical basis for action selection by the agent, is exclusively built from locally available information, which includes:
  - **Vehicle kinematics:** the speed (VS) and acceleration (VA) of the ego vehicle obtained from the vehicle kinematics estimator.
  - **CBR:** the ratio of channel occupancy at the previous frame obtained from the access radio.
  - **Number of neighbor vehicles (NNV):** number of distinct vehicles whose beacons are successfully received in the last 20 frames.
  - **Average Beacon Interval (ABI):** average beacon interval between successively received beacons over all neighbor vehicles, quantified in the unit of frame duration *T*.
  Then, the state of the agent is represented by a 5-tuple vector:
  s=(VS,VA,CBR,NNV,ABI)
5. *Rewards* play a pivotal role in guiding the learning process of the agent. Upon executing an action *a* at time *t*, the agent transits from its current state *s* to a new state $s^{\prime }$, during which it receives a reward $R_{t+1}$. This reward, a critical form of feedback from the environment, is formulated as a function of both the state *s* and the action *a*. The specific structure and implications of this reward mechanism, which fundamentally influences the agent’s behavior, will be elaborated upon in the following section.

### 4.4. Design Principle of Reward Functions

In DRL, the choice between sparse and dense rewards is critical in determining how effectively an agent learns from its environment [30,31]. Sparse rewards are given only when a specific goal or milestone is reached, making them suitable for tasks where the path to the goal is less important than the outcome. However, this approach can lead to slow learning, as the agent receives minimal feedback on its actions. Conversely, dense rewards provide continuous feedback at each time step, guiding the agent with incremental signals that can significantly accelerate learning, especially in tasks requiring ongoing adjustments.

In the context of vehicular beaconing, dense rewards are particularly well-suited because of the need for continuous vehicle awareness to prevent vehicle collisions. Beaconing involves real-time detection and tracking of vehicles, where each decision can immediately impact safety and communication efficiency. By using a dense reward structure, the agent is encouraged to make ongoing improvements in maintaining awareness, optimizing detection and tracking, and managing channel congestion. This continuous feedback loop is crucial in dynamic traffic environments, ensuring that the agent adapts its behavior to minimize risks and maintain optimal performance.

The design of our dense reward function is intricately crafted to address the core challenges in beaconing: awareness in terms of vehicle detection, vehicle tracking, and their minimum failure distances, timeliness, and channel congestion. This function serves as the cornerstone of our DRL framework, meticulously balancing the trade-offs between these objectives to guide the decision-making process of the agent. Our reward structure aims to foster a harmonious integration of these critical aspects by appropriately setting the weight assigned to each reward component. This thoughtful design not only meets the operational requirements of beaconing but also promotes the efficient and sustainable use of resources.

Our reward function consists of five components, i.e., the mDFD, mTFD, MTE, beacon interval, and congestion costs:

(3) $$R_{t+1}=-mdfdCost\left(d\right)-mtfdCost\left(d\right)-mteCost-biCost-cgCost,$$

where each reward component will be addressed as follows:

- **mDFD cost** mdfdCost(d): The mDFD cost is the penalty applied to the distance *d* to the nearest undetected vehicle. Since the ego vehicle is unaware of this undetected vehicle, a short mDFD no greater than the detection distance threshold $\theta_{D}$ poses the most significant safety risk. To reflect this severity, the highest penalty value should be set to its weight $\omega_{DF}$ in (4). Since the risk of V2V collision quickly decreases with the increase in distance between the colliding vehicles, we exponentially decrease the mdfdCost(d) if the distance exceeds the threshold $\theta_{D}$, as follows:
  (4) $$mdfdCost\left(d\right)=\left\{\begin{array}{cc} \omega_{DF}, & 0\leq d\leq \theta_{D};\\ \omega_{DF}\;\cdot \;\exp\left(-\frac{d-\theta_{D}}{\eta \;\cdot \;\theta_{D}}\right), & \theta_{D}<d\leq D_{T}, \end{array}\right.$$
  where parameter η determines the exponential time constant, which controls how quickly the cost decreases as the distance to the colliding vehicle increases.
- **mTFD cost** mtfdCost(d): The mTFD cost is the penalty imposed on the distance *d* to the nearest untracked vehicle. Although this vehicle has already been detected, a short mTFD less than the tracking distance threshold $\theta_{T}$ potentially yields a V2V collision. Similar to the mDFD, if the distance is no greater than $\theta_{T}$, a relatively high constant weight $\omega_{TF}$ is allocated to the mTFD cost; otherwise, it decreases exponentially with the relative distance, as follows:
  (5) $$mtfdCost\left(d\right)=\left\{\begin{array}{cc} \omega_{TF}, & 0\leq d\leq \theta_{T};\\ \omega_{TF}\;\cdot \;\exp\left(-\frac{d-\theta_{T}}{\theta_{T}}\right), & \theta_{T}<d\leq D_{T}. \end{array}\right.$$
- **MTE cost** mteCost: The MTE cost refers to the maximum TE of a tracked neighbor vehicle $v\in V_{G}$, where it is always less than tracking threshold $E_{T}$, as follows:
  (6) $$mteCost=\omega_{TE}\;\cdot \;\max_{\forall v}\left(TE(v)\right),\;\;\;\;TE\left(v\right)\leq E_{T}.$$
  Although it does not incur a risk of collision, the MTE cost can be interpreted as the upper bound of the tracking accuracy of a neighbor vehicle.
- **Beacon interval cost** biCost: The beacon interval cost informs the agent about the elapsed time since its last beacon broadcast, which is crucial for ensuring the freshness of kinematic information updates. It is proportional to the ABI to quantify the beacon aging:
  (7) $$biCost=\omega_{BI}\;\cdot \;ABI.$$
- **Congestion cost** cgCost: The congestion cost encourages the agent to make judicious decisions regarding beacon transmission in relation to both current CBR and TP level, as follows:
  (8) $$cgCost=\omega_{CG}\;\cdot \;P_{TX}\left(p\right),$$
  where congestion weight $\omega_{CG}$ plays a pivotal role when the agent opts for the appropriate action a=(1,p) by discriminating the weights by comparing the current CBR with the CBR threshold $\theta_{G}$, as follows:
  (9) $$\omega_{CG}=\left\{\begin{array}{cc} 0, & \mathrm{if}\;b=0;\\ CBR, & \mathrm{if}\;b=1\;\mathrm{and}\;\mathrm{CBR}\leq \theta_{G};\\ \delta , & \mathrm{if}\;b=1\;\mathrm{and}\;\mathrm{CBR}>\theta_{G}, \end{array}\right.$$
  where parameter δ acts as a penalty constant that significantly raises the congestion weight $\omega_{CG}$ when the CBR exceeds the threshold $\theta_{G}$. This penalty increases the congestion cost, signaling to the agent that the selected TP is causing the CBR to exceed the desirable range of CBR. In addition, TP cost $P_{TX}\left(p\right)$ is proportionally scaled to the TP *p*, as follows:
  (10) $$P_{TX}\left(p\right)=C_{1}+\frac{p-p_{0}}{\Delta p}C_{2},$$
  where $C_{1}$ is the default cost coefficient for beacon transmission, $C_{2}$ the incremental cost coefficient of TP, $p_{0}$ the smallest TP in dBm, and Δp the TP gap.

We notice that the first three awareness components, i.e., mDFD, mTFD, and MTE, are not locally available to the agent. During the training phase of the SAC network, these additional terms are used to calculate the target Q-value, which is then approximated by the critic network. However, these terms are no longer needed in the test phase of the SAC network, as the Q-value predicted by the trained critic network will be used for action-value function approximation.

## 5. Numerical Results

In this section, we provide a detailed analysis of the DRL-JCBRTP framework’s performance through simulations and comparative assessments. We begin by outlining the simulation environment along with the training, testing, and deployment phases in Section 5.1. Section 5.2 covers the parameter settings for our reward components, which are critical for optimizing performance. In Section 5.3, we examine the impact of different NN architectures on beaconing performance. Finally, Section 5.4 presents a comparison of the DRL-JCBRTP scheme with other beaconing schemes to highlight its effectiveness in various vehicular scenarios.

### 5.1. Simulation Framework, Training, Testing, and Deployments

**Figure 5:**
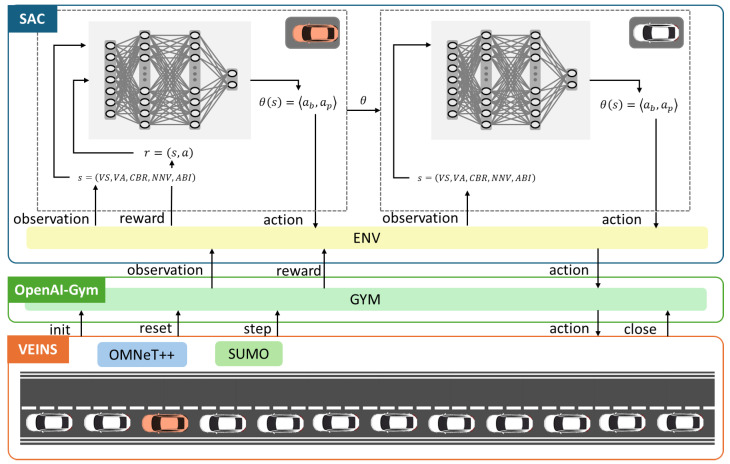
SLMLab-Gym-VEINS simulation framework.

We developed the SLMLab-Gym-VEINS simulation framework to train and evaluate the DRL−JCBRTP policy, as illustrated in Figure 5. This comprehensive setup merges the VEINS simulation framework—which itself is a fusion of the SUMO mobility simulator and the OMNeT++ network simulator—with the OpenAI-Gym framework to support SLMLab [32,33,34,35,36]. Our simulation environment, designed for both training and testing phases, features a 2 km highway segment with multiple vehicle density scenarios. Training was conducted across various vehicle densities and inter-vehicle distances, with vehicles operating under a car-following model. To accurately capture wireless channel characteristics in high-mobility scenarios, the Nakagami-m fading model was employed.

During the training phase, a single agent (the red vehicle in Figure 5) was responsible for policy updates, while all other agents (white vehicles) acted based on this policy. This was facilitated by dual SAC networks: one network updated its weights based on observations from the red vehicle at the beginning of each frame, incorporating recent policy adjustments from the red vehicle’s experiences. Subsequently, the second network in each white vehicle adopted these updated weights and performed inference using its own state information.

Training was conducted over 32,000 frames, with each frame representing a step in the simulation. Each scenario was treated as an episode lasting 160 frames, after which the system randomly selected a new scenario to expose the agent to a diverse set of conditions. This approach helped generalize the policy across different densities and inter-vehicle distances.

In the testing phase, our scenarios were executed using the dual SAC networks exclusively for inference, with no further training. The networks’ beaconing performance was assessed by comparing it to other state-of-the-art schemes across various metrics, including BR, TP, and CBR adaptability. These metrics, which will be discussed in detail later, were used to evaluate the strengths and weaknesses of our approach and to demonstrate its ability to increase mAFD relative to existing methods. This ensures that our networks are robust, efficient, and effective across a variety of densities and inter-vehicle distances.

Regarding the deployment of DRL-JCBRTP in real vehicles, it is important to note that while exploration and exploitation are integral during the training phase, the deployment phase focuses solely on inference. The training phase is conducted using the SLMLab-Gym-VEINS simulation framework, which allows for safe and comprehensive exploration within a simulated environment. In a real-world deployment, the trained NN is integrated into the vehicle’s systems for real-time decision-making based on the learned policies. The NN processes real-time data from the vehicle’s sensors and V2X communications to make quick, accurate decisions regarding beaconing strategies. This allows the vehicle to adapt to dynamic traffic conditions and optimize communication efficiency without the need for further exploration, which mitigates the high costs and risks associated with exploration in real environments. Through this approach, DRL-JCBRTP enhances the vehicle’s ability to maintain robust and reliable communication, ultimately contributing to improved traffic management and collision avoidance.

### 5.2. Weight and Parameter Setting of Our Reward Components

The parameters used in our simulation during the training and testing were selected to represent the radio channel in the Highway 101 Bay Area [37]. Table 1 lists the detailed parameter values in our simulation.

Determining the appropriate weights in the reward function is a crucial aspect of our approach. Unfortunately, to the best of our knowledge, there is no universally accepted method for determining optimal hyperparameters, including the weights used in the reward function, within AI research. Due to this, we conducted extensive experiments to explore different combinations of weights, aiming to identify a near-optimal set of values that best balances the competing objectives inherent in our reward function.

To ensure safe driving, the tracking error threshold $E_{T}$ is set to 1.5 m, as recommended by the National Highway Traffic Safety Administration [2]. The detection ($\theta_{D}$) and tracking threshold ($\theta_{T}$) distances are set based on the driver’s perception-to-reaction time, which ranges from 1.5 s to 3.0 s for vehicles driving in the opposite direction [38]. By considering the maximum reaction time and speed limit, we set $\theta_{T}$ to 90 m. To allow for the earliest possible detection of an oncoming vehicle, the detection threshold distance $\theta_{D}$ is set to twice the tracking threshold distance, resulting in $\theta_{D}=2\;\cdot \;\theta_{T}=180$ m. Additionally, we assigned a higher weight to $\omega_{DF}$ compared to $\omega_{TF}$, because the mDFD is more critical for safety than the mTFD. As the distance approaches the transmission range $D_{T}=500$ m, the impact of detection cost diminishes, which is addressed by setting η=0.5 to satisfy $mdfdCost\left(D_{T}\right)\leq E_{T}$.

For the CBR threshold, we set $\theta_{G}=0.7$, which acts as a critical point where congestion costs begin to significantly impact network performance. This value was selected to strike a balance between preventing network congestion and ensuring effective communications between vehicles. We set the small incremental cost coefficient $C_{2}=0.01$ to introduce a gradual increase in the cost associated with different TP levels, ensuring that higher TPs are not excessively penalized. This decision is based on the fact that vehicles typically have less severe restrictions on TP compared to mobile devices, allowing for greater flexibility in power usage without incurring significant cost implications. In addition, we established the beacon transmission cost coefficient $C_{1}=5$ to balance the reward components, particularly when the CBR is below the threshold $\theta_{G}$. This strategy helps manage the overall cost structure, ensuring that beaconing actions remain both cost-effective and efficient, while still maintaining optimal network performance.

The remaining SAC hyperparameters, including the learning rate and MLP structures, were meticulously fine-tuned through extensive experimentation to achieve an optimal balance between beacon interval costs and congestion costs. Specifically, we tested different learning rates, starting with 0.005 and refining it to 0.0003, to find a balance between convergence speed and stability. Additionally, we evaluated various MLP configurations consisting of 64 and 256 neurons to identify the best performance in terms of learning efficiency and model accuracy. After extensive testing, we ultimately selected a configuration with 512 hidden units, as discussed in Section 5.3, which provided the most robust performance across multiple scenarios. These adjustments were crucial for ensuring that our model effectively manages network resources, particularly under varying traffic conditions, without compromising overall system performance.

### 5.3. Impacts of DRL-JCBRTP NN Architectures on Beaconing Performance

In Section 4.2, we introduced the SAC algorithmthe SAC algorithm is introduced as a model-free, off-policy DRL approach that utilizesemploys both actor and critic NNs to optimize beaconing performance in dynamic vehicular environments. The weights of both the SAC networks were initialized using the Xavier initialization method, which is effective in maintaining the variance of activations across layers, ensuring stable learning [39]. In our approach, theThe critic NN is structured as an MLP with two hidden layers, each consisting of 512 neurons, utilizing the ReLU activation function. To optimize the networks, we employed the Adam optimizer, which combines the benefits of adaptive learning rates (as in RMSProp) with the momentum technique to enhance convergence speed and stability [40]. Additionally, the learning rate was scheduled using Polyak averaging, which dynamically adjusts the learning rate to maintain efficient and stable training [41]. In this section, we evaluate the performance of four distinct actor NN architectures within the SAC framework, aiming to optimize beaconing performance in dynamic vehicular environments: MLP, one-layer, two-layer, and three-layer LSTMs.

The first actor network mirrors the critic network, employing an MLP with identical architecture and parameters. For the LSTM-based actor networks, each layer is configured with 256 hidden units and a sequence length of 10, focusing on learning and predicting sequences of beacon intervals. We then explore one-layer, two-layer, and three-layer LSTM configurations, with each additional layer following the same setup. This comprehensive comparison seeks to identify the most effective architecture for the actor NN in the SAC algorithm, with a focus on performance and the ability to learn complex sequences. We trainedIt is important to note that all these networks were trained using prioritized replay memory and the SAC parameters listed in Table 1.

**Figure 6:**
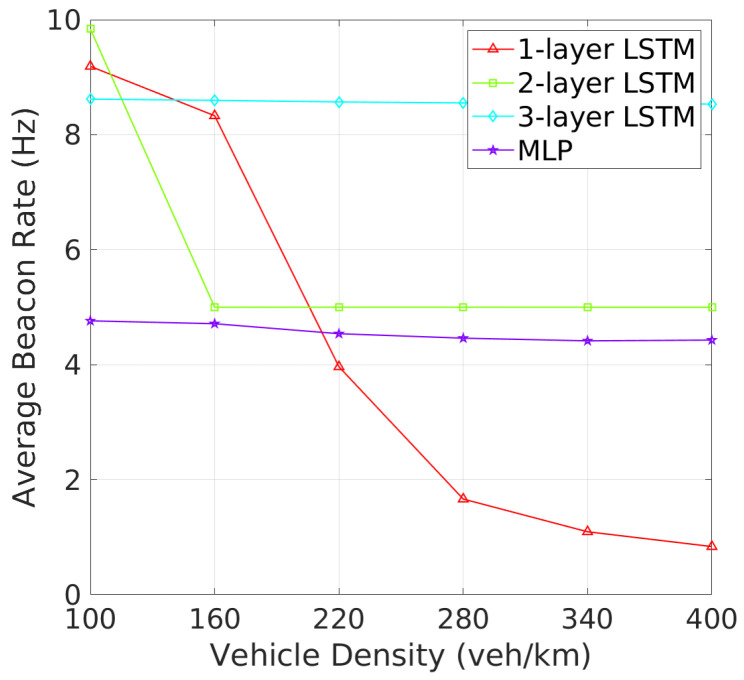
Beacon rate vs. vehicle density.

**Figure 7:**
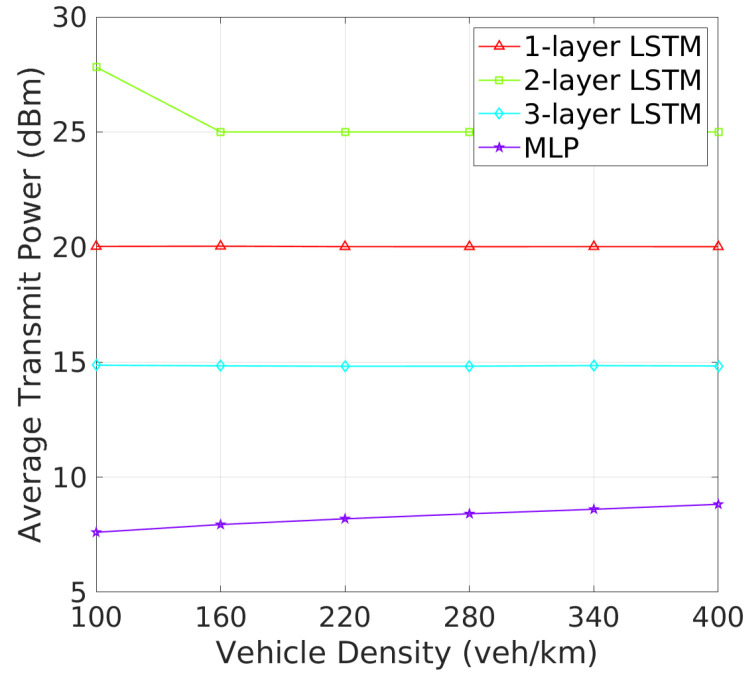
Transmit power vs. vehicle density.

**Beacon Rate and Transmit Power**: The analysis of BR and TP as vehicle density increases reveals critical insights into how each architecture adapts to varying traffic conditions. As shown in Figure 6, one-layer and two-layer LSTM models exhibit significant adaptability by adjusting their BRs in response to increased vehicle density. This dynamic response is likely a mechanism to manage channel congestion in denser environments. On the other hand, three-layer LSTM maintains a stable BR similar to the MLP, the 3-layer LSTM and MLP maintain nearly constant beacon rates, indicating a potentially less adaptive or more conservative strategy under high-density conditions. Figure 7 shows that the MLP model gradually increases its TP with rising vehicle density, which could worsen channel congestion. In addition, one-layer and three-layer LSTM models keep their TP nearly constant, suggesting their challenges in effective TP policy learning. In contrast, the TP of two-layer LSTM model starts at 28 dBm, decreasing and stabilizing at around 25 dBm as vehicle density increases. These results show that two-layer LSTM adapts its TP more effectively, better maintaining an optimal CBR and reducing beacon collisions, making it more efficient in varying traffic conditions.In terms of TP, as illustrated in Figure 7, the 2-layer LSTM and MLP models demonstrate a clear pattern of adjusting TP with increasing vehicle density, which suggests these models are balancing communication range with the need to reduce interference. Meanwhile, the 1-layer and 3-layer LSTM models show less variation in TP, which may suggest a fixed strategy irrespective of vehicle density.

**Figure 8:**
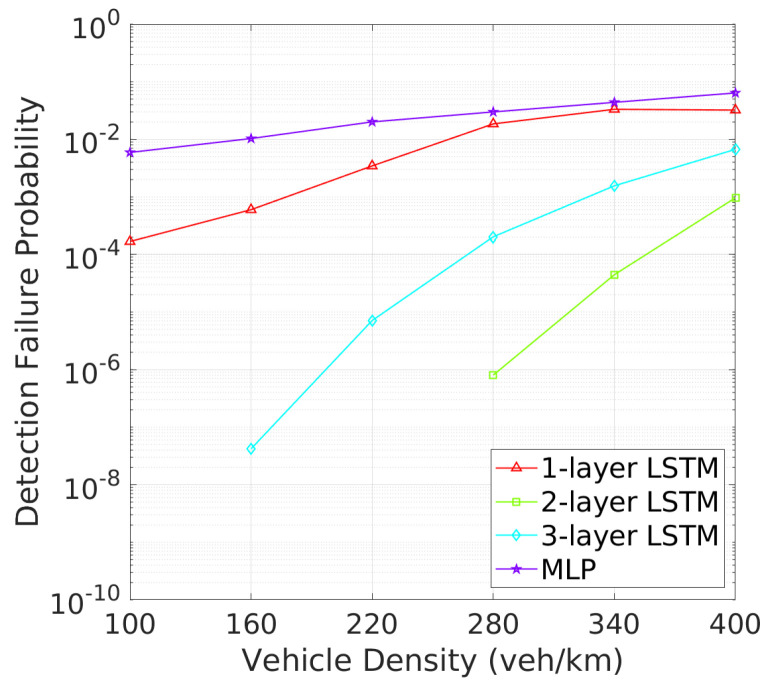
DFP vs. vehicle density.

**Figure 9:**
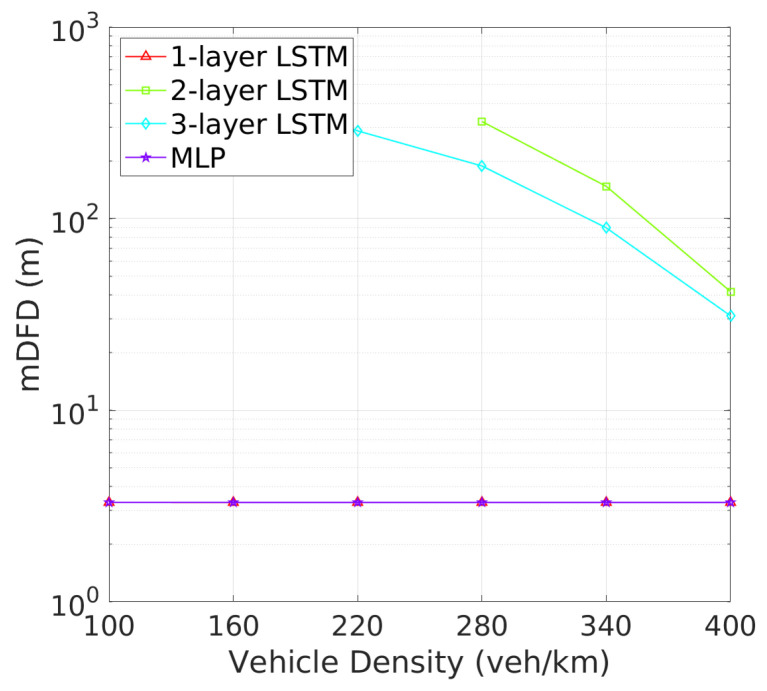
mDFD vs. vehicle density.

**Vehicle Detection Performance**: Next, we turn to the detection failure metrics, where the 2-layer LSTM model continues to demonstrate superior performance. As depicted in Figure 8 illustrates that two-layer LSTM consistently maintains a low detection failure probability (DFP), with only a minimal increase at higher vehicle densities, demonstrating its robust detection capabilities. In comparison, three-layer LSTM, though more stable than one-layer LSTM and MLP, exhibits a higher DFP than two-layer LSTM., the detection failure probability (DFP) for the 2-layer LSTM remains extremely low across all vehicle densities, with only a slight increase at higher densities. Figure 9 further underscores the superiority of two-layer LSTM, which consistently achieves a significantly larger mDFD than the other models. While three-layer LSTM shows improvement, it still falls short, particularly in high-density scenarios. These results clearly indicate that two-layer LSTM is the most effective at maintaining detection reliability and distance across varying vehicle densities.This low DFP correlates with a high mDFD, as shown in Figure 9, where two-layer LSTM outperforms the other models, particularly at lower densities. The 1-layer LSTM shows a rapid degradation of DFP as vehicle density increases. The 3-layer LSTM and MLP models, however, maintain relatively constant but higher DFP and lower mDFD, indicating a less adaptive approach to varying traffic conditions.

**Figure 10:**
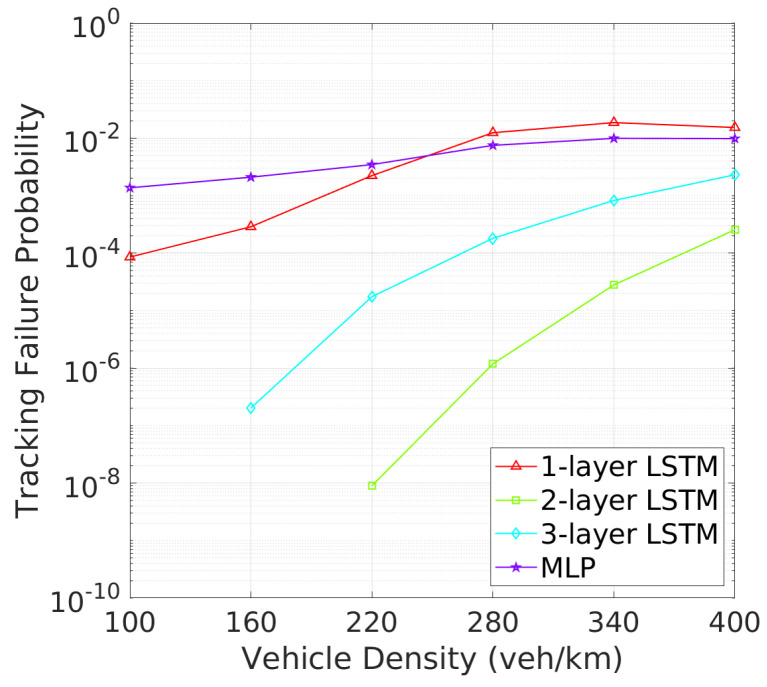
TFP vs. vehicle density.

**Figure 11:**
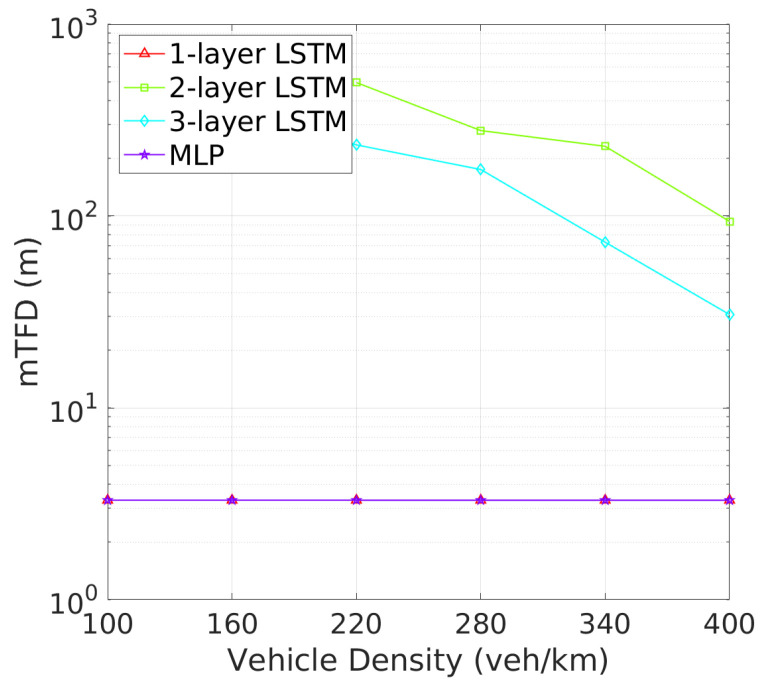
mTFD vs. vehicle density.

**Vehicle Tracking and Awareness Performance**: This trend continues when evaluating tracking failure probability (TFP) in Figure 10 and mTFD in Figure 11. Two-layer LSTM again outperforms other architectures, with the lowest TFP and highest mTFD across all vehicle densities. Two-layer LSTM, while demonstrating improved performance over one-layer LSTM and MLP, still cannot match two-layer LSTM, particularly in higher vehicle density scenarios where effective tracking is critical. As illustrated in Figure 10, the tracking failure probability (TFP) for the 2-layer LSTM remains consistently low, only slightly increasing at higher vehicle densities. This consistent performance translates into superior mTFD, as evidenced in Figure 11, where the 2-layer LSTM maintains a notable mTFD even at the highest vehicle densities. Conversely, while the 1-layer LSTM starts off competitive, it experiences a rapid increase in TFP as density grows. Three-layer LSTM and MLP models, while stable, exhibit higher TFPs and consequently maintain lower mTFD values, suggesting that their tracking capabilities may be more compromised under dense traffic conditions.

**Figure 12:**
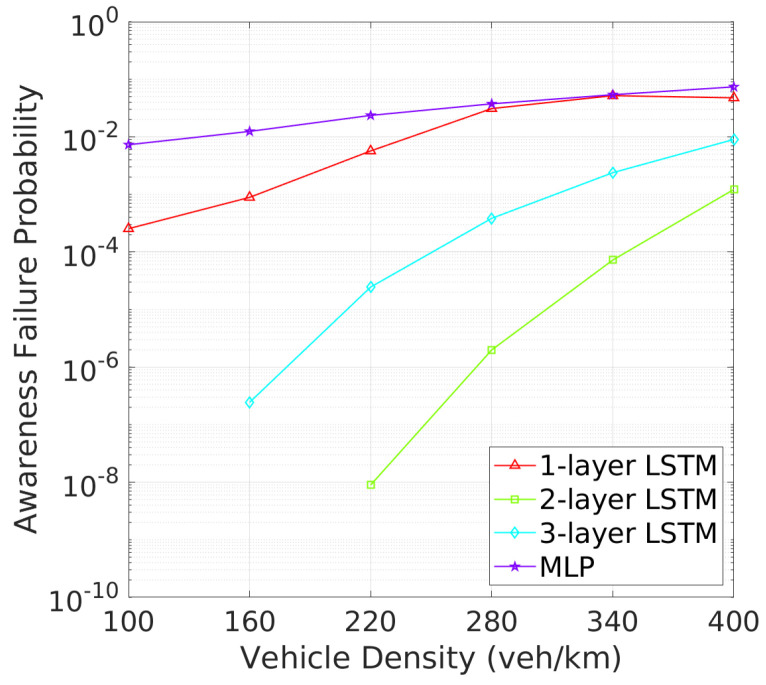
AFP vs. vehicle density.

**Figure 13:**
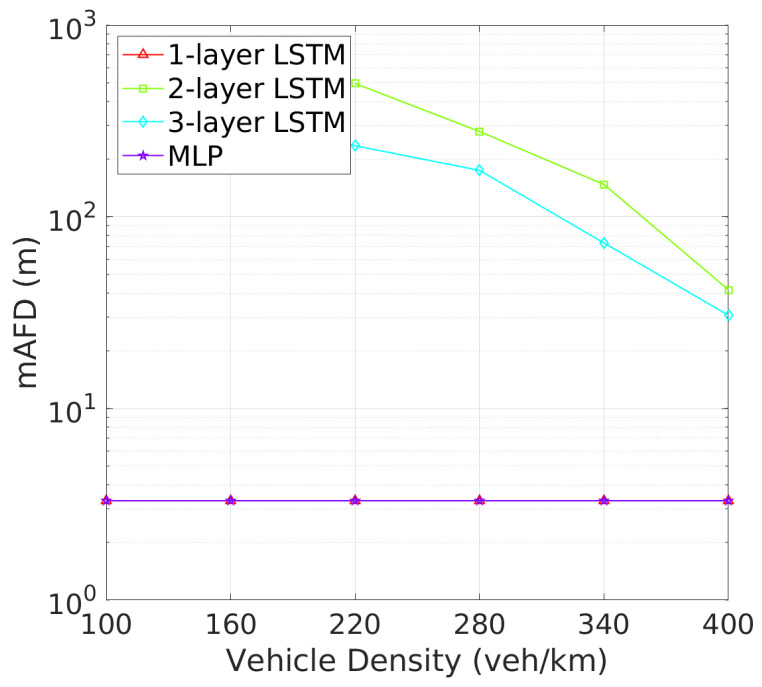
mAFD vs. vehicle density.

Similarly, Figure 12 and Figure 13 reveal that two-layer LSTM maintains the lowest awareness failure probability (AFP) and mAFD, making it the most reliable model for situational awareness in dynamic vehicular environments, while the added complexity of three-layer LSTM does not significantly enhance tracking or awareness capabilities, which remain slightly inferior to those of the two-layer configuration. Finally, the awareness failure probability (AFP) in Figure 12 and mAFD metrics in Figure 13 further highlight the robustness of the 2-layer LSTM. The AFP for the 2-layer LSTM is the lowest among four NN archtectures, showcasing its robustness in maintaining situational awareness across varying vehicle densities. This leads to the highest mAFD, where the 2-layer LSTM clearly outperforms others, maintaining significantly greater mAFD even under high-density conditions.

**Figure 14:**
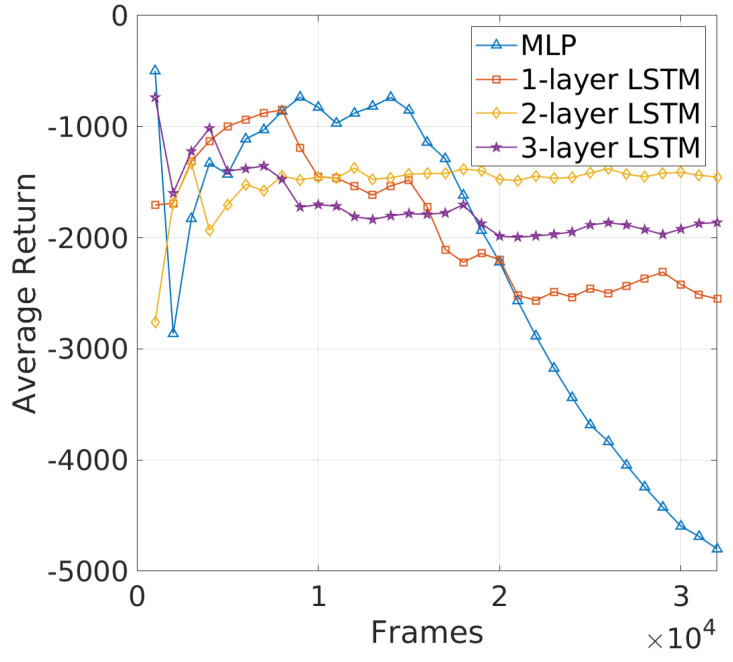
Learning curve of DRL−JCBRTP.

**Challenges in Learning Efficiency of DRL−JCBRTP**: The learning curves in Figure 14 provide a clear comparison of the average return for different NN architectures. Notably, two-layer LSTM shows a relatively stable and high average return, indicating robust learning and effective adaptation to the beaconing problem. Three-layer LSTM, while more complex, stabilizes at a lower return than two-layer LSTM, suggesting that the added complexity does not significantly enhance learning and may even introduce challenges in optimization. The MLP and one-layer LSTM models, on the other hand, exhibit considerable fluctuations and ultimately deteriorate in performance, particularly the MLP, which fails to maintain effective learning and collapses as training progresses. These results highlight the advantage of using a two-layer LSTM in capturing the temporal dependencies crucial for beaconing, where the simple MLP struggles due to its inability to effectively handle time-series data. To summarize, the 2-layer LSTM consistently delivers superior performance across all metrics, making it the most reliable architecture for dynamic vehicular environments. These results firmly establish the 2-layer LSTM as the preferred DRL−JCBRTP for performance comparisons in the following section.

In the context of V2X beaconing, unlike in more controlled environments such as robotics, the relationship between an agent’s actions and the resulting rewards can be less direct due to the stochastic nature of the environment. First, even if the ego vehicle transmits a beacon, some neighbor vehicles may fail to receive it due to wireless channel impairments, leading to degraded detection and tracking performance. Second, the ego vehicle’s contribution to the CBR is inversely proportional to the number of vehicles within its transmission range. This means that the impact of any single action on the overall network performance is diluted when there are many vehicles, making it harder for the agent to learn an effective action policy. These factors reduce the correlation between actions and rewards, complicating the training process and potentially leading to suboptimal policy learning.

We hypothesize that these challenges suggest the loss function in the training process might be represented as a highly multi-modal nonlinear function in the weight space, making optimization particularly difficult. While we applied advanced techniques such as Xavier initialization to mitigate exploding and vanishing gradients, the ADAM optimizer for efficient convergence, and the Polyak averaging to stabilize the learning process, the optimality of the policies exhibits significant variability, indicating that the learned policies may not consistently converge to the true optimal policy. As expected, validating this hypothesis requires further research, as additional studies are needed to thoroughly investigate and confirm the underlying complexities of the objective function in such dynamic environments. Nonetheless, the use of LSTM networks, especially two-layer LSTM, proves to be more effective than the MLP, as evidenced by the superior performance in maintaining higher awareness levels, confirming the importance of considering temporal dependencies in such dynamic environments.

### 5.4. Performance Comparison with Other Beaconing Schemes

This section presents a comparative analysis of the DRL-JCBRTP scheme against three distinct beaconing schemes, each with its unique method for BR and/or TP control. The schemes compared are as follows:

- **BFPC** [12]: a game-theoretic approach focusing on CBR to determine the BR and TP.
- **MDPRP** [13]: a DRL approach that adjusts its beaconing parameters based primarily on CBR.
- **MAB^2^** [17]: a BRC scheme that provides guaranteed tracking accuracy with optimal channel congestion in unreliable wireless channels.

Each of these schemes brings a different perspective to the challenges of BR and TP control, enabling a fair assessment of our DRL−JCBRTP scheme.

**Figure 15:**
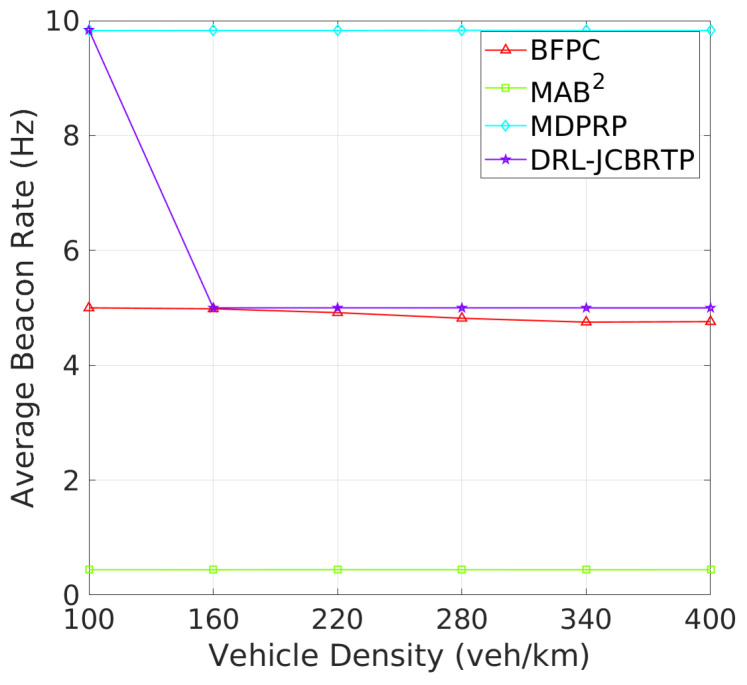
Beacon rate vs. vehicle density.

**Figure 16:**
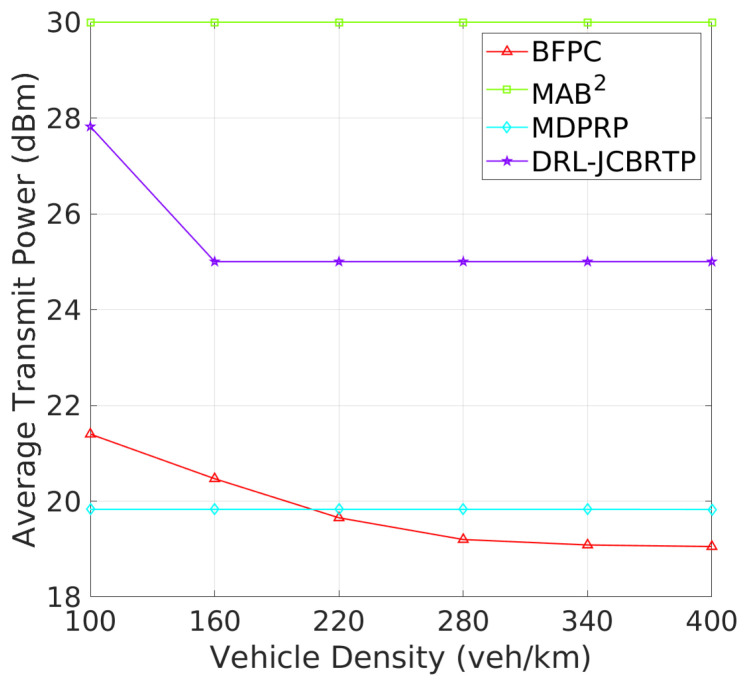
Transmit power vs. vehicle density.

**Beacon Rate and Transmit Power**: We begin by evaluating the ability to adaptively adjust the beaconing parameters depending on vehicle density. Figure 15 shows the average rates of beacon broadcast for different vehicle densities ranging from 100 to 400 veh/Km. As the vehicle density increases, we observe two distinct patterns of BRC: (1) The MAB^2^, BFPC, and MDPRP schemes keep consistent BRs regardless of vehicle density; and (2) the DRL−JCBRTP scheme decreases its BR with vehicle density, demonstrating its adaptability to control the channel congestion. Figure 16 shows the average beacon TP against vehicle density. Both our DRL−JCBRTP and BFPC schemes adaptively adjust their TPs, while the MDPRP and MAB^2^ schemes maintain nearly constant TPs. These results demonstrate the DRL−JCBRTP scheme’s ability to jointly optimize BR and TP, significantly improving the beaconing performance.

**Figure 17:**
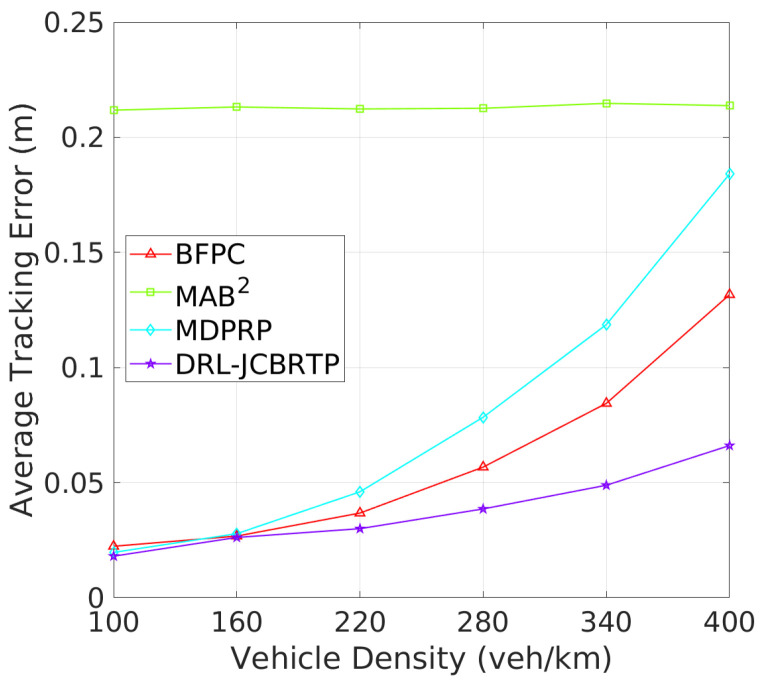
Average TE vs. vehicle density.

**Figure 18:**
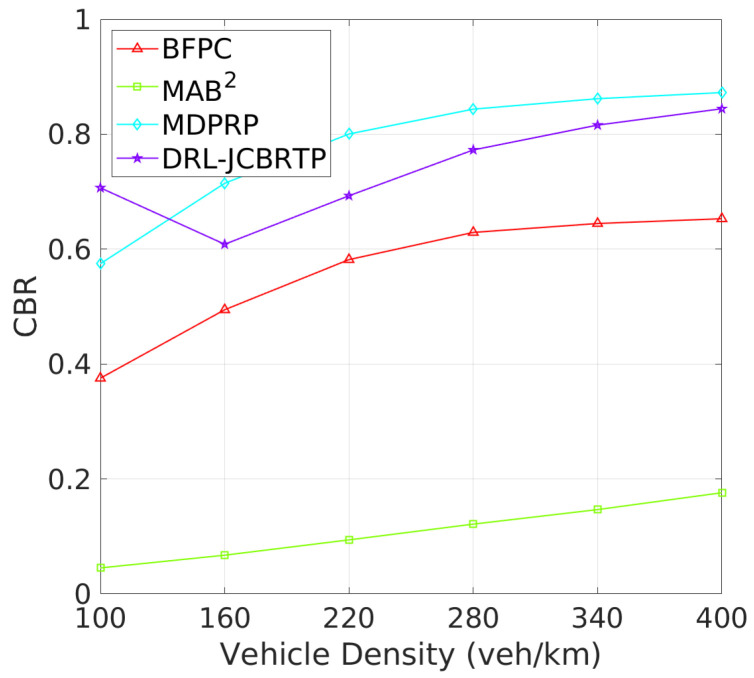
CBR vs. vehicle density.

**Tracking Errors and CBRs**: Next, we assess the overall beaconing performance for different vehicle densities. Figure 17 compares the average TEs of beaconing schemes. While the average TE of the MAB^2^ scheme is almost constant regardless of vehicle density, the average TEs of the other beaconing schemes rapidly grow with vehicle density. Our DRL−JCBRTP scheme achieves the best tracking accuracy among these schemes across all vehicle densities. Figure 18 shows the channel busy ratio of these schemes against vehicle densities. The MAB^2^ scheme is theoretically proven to optimize the CBR while achieving a guaranteed tracking performance. We also observe that the CBRs of the other beaconing schemes gradually increase with vehicle density. However, at 100 veh/Km, our DRL−JCBRTP scheme shows the highest CBR among all these schemes due to the increase in both BR and TP. Overall, the DRL−JCBRTP scheme stands out by achieving the best tracking accuracy and efficient channel utilization across all vehicle densities.

**Figure 19:**
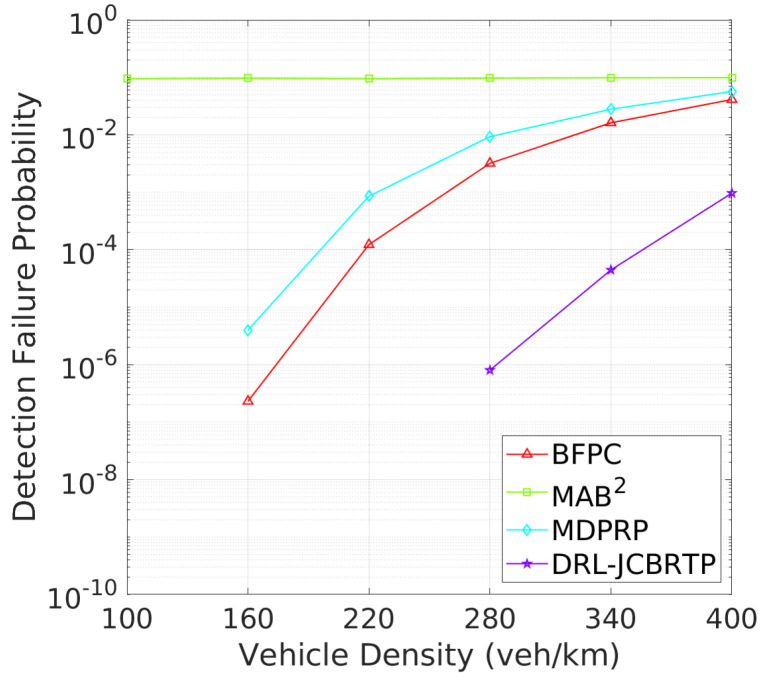
DFP vs. vehicle density.

**Figure 20:**
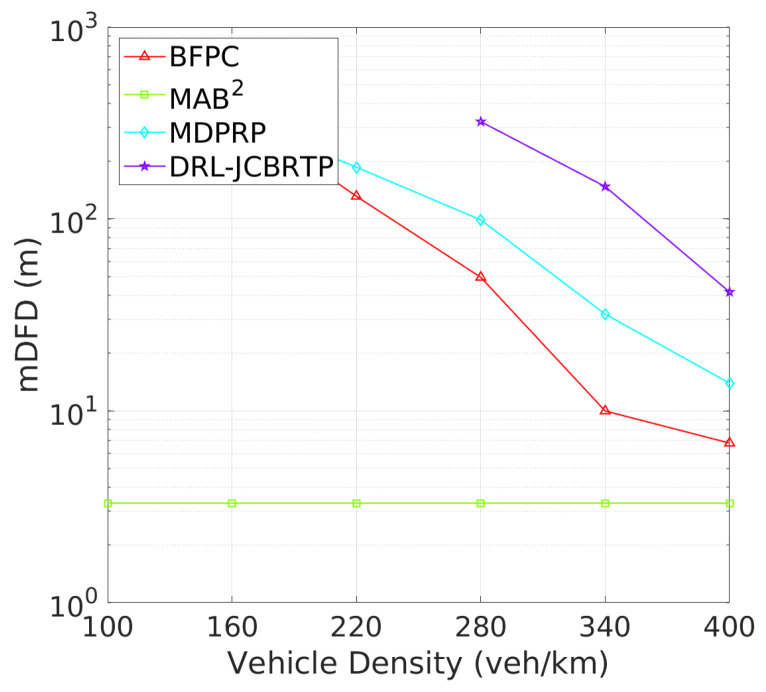
mDFD vs. vehicle density.

**Vehicle Detection Performance**: Following the beaconing performance analysis, we examine the detection failure performance of beaconing schemes. Figure 19 shows the DFP against vehicle density. The DRL-JCBRTP scheme consistently achieves the lowest DFP across all vehicle densities, demonstrating its ability to minimize the number of undetected vehicles. In contrast, the BFPC and MDPRP schemes exhibit higher DFPs, while the MAB2 scheme maintains the highest constant DFP. Figure 20 illustrates the minimum detection failure distance against vehicle density. The MAB^2^ scheme shows a near-zero mDFD across all vehicle densities, indicating its high risk of V2V collision due to undetected nearby vehicles. As vehicle density increases, mDFD tends to decrease due to increased channel congestion. Notably, our DRL-JCBRTP scheme shows the longest mDFD compared to other schemes. These results demonstrate the superior performance of the DRL-JCBRTP scheme in terms of both DFP and mDFD. By optimizing the detection failure performance across all vehicle densities, the DRL-JCBRTP scheme can significantly reduce the risk of V2V collisions caused by undetected nearby vehicles, thereby enhancing the overall driving safety.

**Figure 21:**
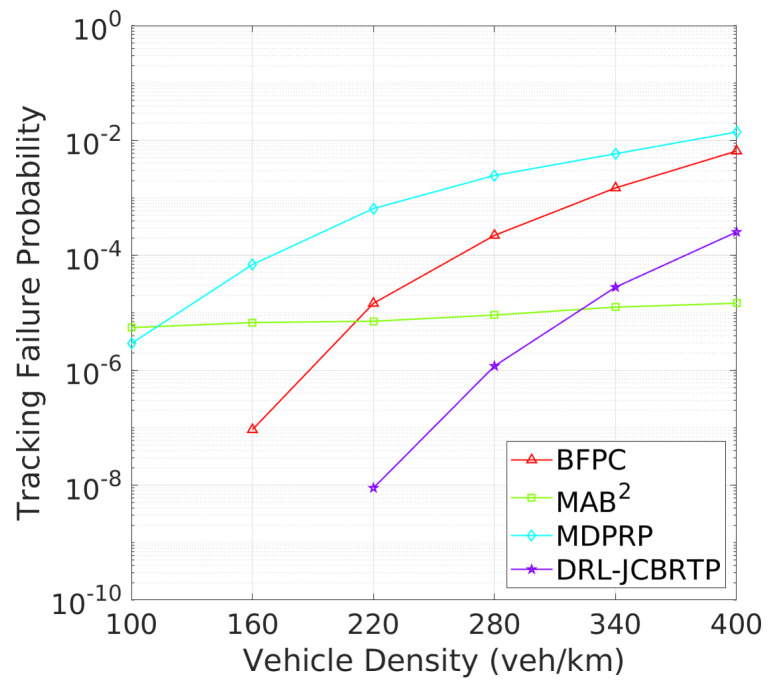
TFP vs. vehicle density.

**Figure 22:**
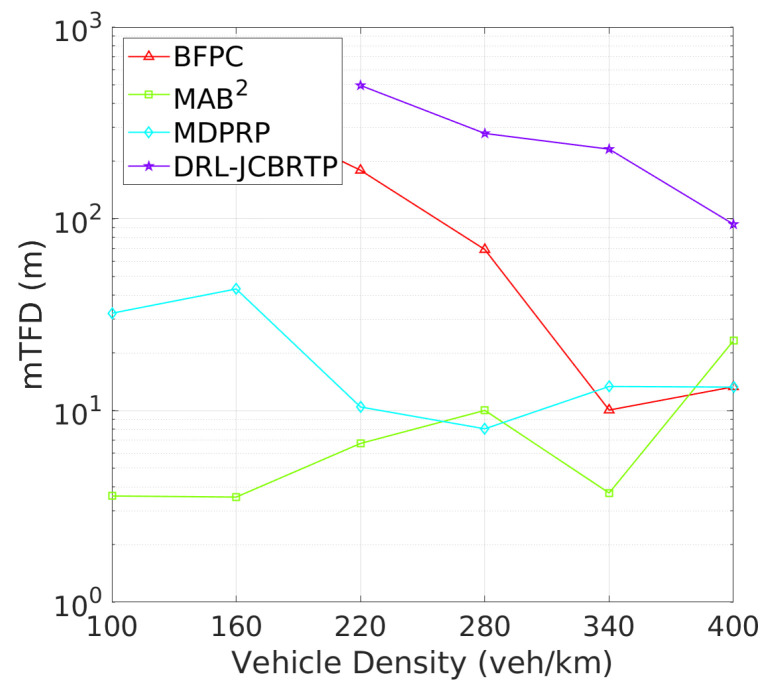
mTFD vs. vehicle density.

**Vehicle Tracking Performance**: The analysis continues with tracking failure performance of beaconing schemes. Figure 21 shows the TFP for different vehicle densities. The DRL-JCBRTP scheme achieves the lowest TFP for low to moderate vehicle densities. On the other hand, the MAB^2^ scheme shows the best TFP performance at high vehicle densities due to its low CBR, while the BFPC and MDPRP schemes exhibit the highest TFPs in moderate to high vehicle densities. Figure 22 illustrates the minimum tracking failure distance against vehicle density. The DRL-JCBRTP scheme shows the longest mTFD among all beaconing schemes, indicating its superior tracking performance. The MAB^2^ scheme, however, consistently shows the lowest mTFD, underscoring its limitations in tracking efficiency. These results highlight the effectiveness of the DRL-JCBRTP scheme in both minimizing TFP and maximizing mTFD. This comprehensive optimization reduces the likelihood of V2V collisions from untracked vehicles, thus improving the overall safety of driving conditions.

**Figure 23:**
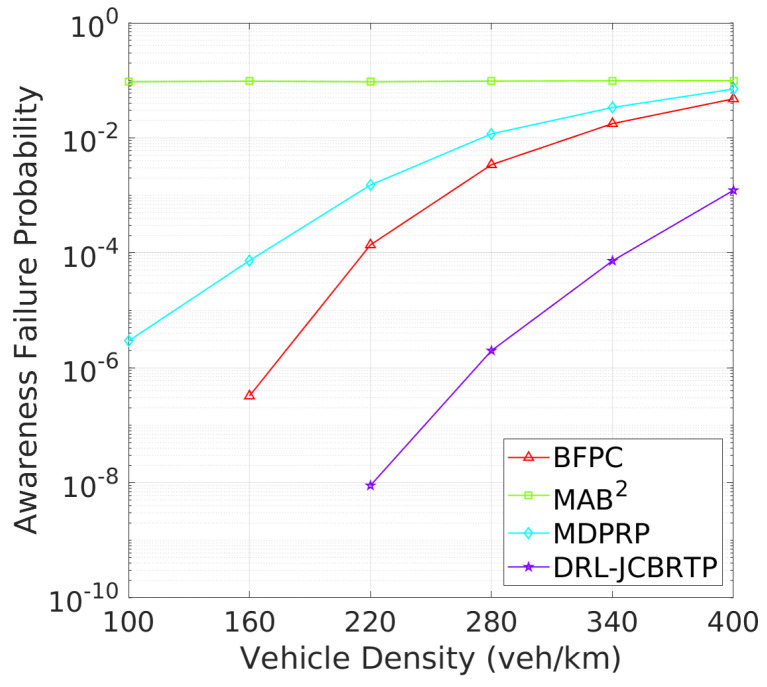
AFP vs. vehicle density.

**Figure 24:**
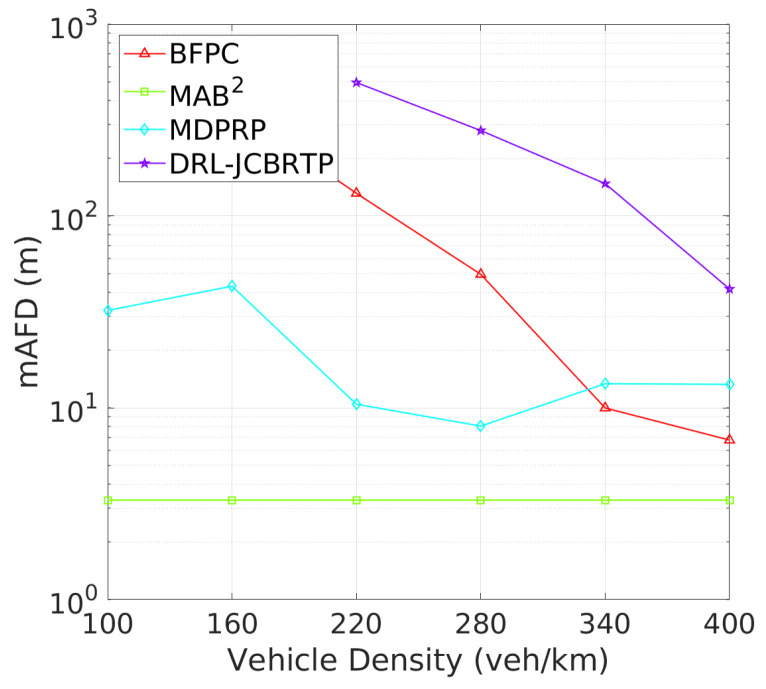
mAFD vs. vehicle density.

**Vehicle Awareness Performance**: Finally, we analyze the awareness failure performance of beaconing schemes, where awareness failure is represented by the union of detection and tracking failures. Figure 23 shows the AFP against vehicle densities. In comparison with Figure 19 and Figure 21, the AFPs of DRL-JCBRTP, BFPC, and MDPRP schemes are primarily determined by their TFPs, whereas the AFP of the MAB^2^ scheme is influenced more by its DFP. Figure 24 illustrates the mAFD against vehicle density. Compared with Figure 20 and Figure 22, it is evident that mTFDs are the dominant factors determining mAFDs of MAB^2^, BFPC, and MDPRP schemes, while both mDFD and mTFD contribute to the AFP of our DRL−JCBRTP scheme. To conclude, these numerical results underscore the comprehensive effectiveness of the DRL-JCBRTP scheme in enhancing vehicle awareness by simultaneously minimizing AFP and maximizing mAFD, ultimately improving driving safety.

## 6. Conclusions

We presented the DRL−JCBRTP framework, a novel deep reinforcement learning approach for beaconing that dynamically adjusts BR and TP to enhance cooperative vehicle awareness. By interpreting awareness as a multi-faceted concept involving vehicle detection, tracking, and safety distances, our DRL framework introduces innovative reward functions to increase the minimum awareness failure distance, balancing both the vehicle’s internal state and external influences. Our SLMLab-Gym-VEINS simulations demonstrate that the DRL-JCBRTP scheme outperforms existing beaconing schemes by minimizing awareness failure probability and maximizing awareness distance. These results highlight its capability to improve cooperative awareness in vehicular environments, consequently enhancing driving safety.

While the DRL−JCBRTP framework has demonstrated significant improvements in cooperative vehicle awareness across a range of traffic densities and inter-vehicle distances, its robustness in handling a wider variety of real-world scenarios—such as different terrains, weather conditions, and other environmental factors—remains an open question. Future research will focus on extending the versatility of the DRL-JCBRTP scheme by training it on more diverse datasets that encompass these variables. This effort will aim to further enhance the model’s adaptability and effectiveness in real-world applications, ensuring that it can maintain high levels of performance even in conditions that were not part of the initial training environment. By addressing these challenges, we hope to develop a more comprehensive solution capable of reliably supporting V2X communications in a broad array of driving scenarios. 

## Figures and Tables

**Table 1:** Simulation parameters.

General Parameters	Value	Unit
Road length	2	km
Vehicle density	100, 160, 220, 280, 340, 400	veh/km
Vehicle speed	30	m/s
Inter-vehicle distance	40, 60, 80	m
Channel frequency	5.89	GHz
Data rate	6	Mbps
Transmit power	0, 5, 10, 15, 20, 25, 30	dBm
Receiver sensitivity	−95	dBm
Message size	400	Bytes
Frame duration (*T*)	100	ms
**Reward Function Parameters**	**Value**	**Unit**
$\omega_{DF}$	48	
$\omega_{TF}$	12	
$\omega_{TE}$	1	
$\omega_{BI}$	7	
$E_{T}$	1.5	m
$\theta_{D}$	180	m
$\theta_{T}$	90	m
$D_{T}$	500	m
η	0.5	
δ	3	
$\theta_{G}$	0.7	
$C_{1}$	5	
$C_{2}$	0.01	
$p_{0}$	0	dBm
Δp	5	dBm
**SAC Parameters**		**Value**
Weight initialization		random
Optimizer		Adam
Learning rate		$3\times 10^{-4}$
Batch size		512
γ		0.99

## Data Availability

The original contributions presented in the study are included in the article, further inquiries can be directed to the corresponding author.
